# Repeatability of exhaled nitric oxide measurements in patients with COPD

**DOI:** 10.1111/j.1475-097X.2010.00975.x

**Published:** 2011-01

**Authors:** Annamari Rouhos, Annette Kainu, Päivi Piirilä, Seppo Sarna, Ari Lindqvist, Jouko Karjalainen, Anssi R A Sovijärvi

**Affiliations:** 1 Department of Medicine, Division of Respiratory Diseases, Helsinki University Central HospitalHelsinki, Finland; 2 Division of Clinical Physiology and Nuclear Medicine, Laboratory Department, Helsinki University Central HospitalHelsinki, Finland; 3 Department of Public Health, University of HelsinkiHelsinki, Finland; 4 Department of Medicine, Research Unit for Pulmonary Diseases, Clinical Research Institute Ltd, Helsinki University Central HospitalHelsinki, Finland; 5 Institute of Military MedicineHelsinki, Finland

**Keywords:** chronic obstructive pulmonary disease, eosinophilic inflammation, fixed bronchial obstruction, fractional exhaled nitric oxide, reproducibility

## Abstract

The assessment of the presence of eosinophilic airway inflammation may help in predicting the steroid response in subjects with respiratory symptoms. Unlike patients with asthma, only a subset of patients with chronic obstructive pulmonary disease (COPD) benefits from steroid treatment. Fractional exhaled nitric oxide (FENO) is a useful surrogate marker for eosinophilic airway inflammation, but data on the repeatability of FENO measurements in COPD needed for the assessment of significant change are insufficient. The aim of this study was to assess the short-term repeatability of FENO measurement in subjects with moderate to very severe chronic airway obstruction compared to that in healthy subjects. We studied 20 patients with stable COPD and 20 healthy subjects, and determined FENO (flow rate 50 ml s^−1^) three times: at baseline, 10 min and 24 h after baseline. Spirometry was performed on the first study day after the FENO measurements. The median FENO concentration in patients with COPD was 15·6 ppb, and in healthy subjects, 15·2 ppb. The coefficient of variation (CoV) for 24-h measurements was 12·4% in COPD patients, and 15·9% in healthy subjects. Among COPD patients with global initiative for chronic obstructive lung disease stage 2 disease, the CoV was 13·7%, and among those with stage 3–4 disease, 10·5%. The findings indicate that the short-term repeatability of FENO measurement in patients with moderate to very severe COPD is equally good as in healthy subjects. A change in FENO exceeding 24% is likely to reflect a minimum measurable change in COPD.

## Introduction

Chronic obstructive pulmonary disease (COPD) is characterized by airflow limitation which is not fully reversible and is usually progressive. Airway inflammation in COPD is dominated by neutrophils and is generally unresponsive to inhaled steroids. Eosinophilia is present in a subset of patients and is often more pronounced during exacerbations (Sutherland & Martin, 2003). COPD patients with frequent exacerbations have been shown to benefit from inhaled steroids (Rabe *et al.*, 2007).

Fractional exhaled nitric oxide (FENO) is considered a reliable surrogate marker for eosinophilic airway inflammation and has proved useful in the diagnosis of asthma (Taylor *et al.*, 2006). FENO levels in COPD vary from low to elevated (Barnes *et al.*, 2006), likely reflecting the heterogeneity of the disease and the FENO-reducing effect of smoking. However, differential diagnosis between asthma and COPD is not always clear cut; the clinical course of asthma sometimes leads to fixed airway obstruction, and partial reversibility may be present in COPD. Partial reversibility has been reported to associate with increased FENO and sputum eosinophilia (Papi *et al.*, 2000), and the presence of eosinophilic airway inflammation in COPD, as verified either by induced sputum or elevated FENO, has been shown to correlate with the steroid response (Brightling *et al.*, 2000; Zietkowski *et al.*, 2005; Leigh *et al.*, 2006; Kunisaki *et al.*, 2008). A response to steroids may result in an improvement of symptoms and lung function and in a reduction of exacerbation frequency (Rabe *et al.*, 2007), making the identification of potential steroid responders important in clinical practice.

The repeatability of FENO measurements has been proven to be good in healthy subjects (Ekroos *et al.*, 2000; Kharitonov *et al.*, 2003), in both non-smokers and smokers (Bohadana *et al.*, 2008), in patients with asthma (Ekroos *et al.*, 2002; Kharitonov *et al.*, 2003) and in subjects with respiratory symptoms (Ekroos *et al.*, 2002). Good repeatability has been reported also in patients with COPD (Brindicci *et al.*, 2005; Bhowmik *et al.*, 2005; de Laurentiis *et al.*, 2008). However, severe obstruction could impair the repeatability, because the requirement of constant respiratory flow for at least 6 s might not be easy to fulfil; these studies have included only a small number of such patients.

The aim of this study was to assess the short-term repeatability of FENO in subjects with moderate to very severe chronic obstructive pulmonary disease compared to that in healthy subjects.

## Methods

### Study population

Twenty patients with previously diagnosed COPD were recruited from the outpatient department of the Division of Respiratory Diseases and from the Research Unit for Respiratory Diseases of the Helsinki University Central Hospital. COPD diagnosis was verified with information available from subjects’ hospital records (mean 5 years since diagnosis, range 1–12 years). All subjects had forced expiratory volume in 1 s (FEV1) <80% of predicted (Viljanen *et al.*, 1982) and the ratio of FEV1 to forced vital capacity (FVC) of <0·7. The chosen inclusion criteria required the subjects to be ex-smokers (mean 52 pack-years, range 20–100) who had stopped smoking at least 1 year ago (mean 6 years, range 1–21 years) and who were clinically stable with no changes in their medication during the preceding 4 weeks. The subjects continued their regular medication, with the exception of short-acting beta-agonists (SABA), which were withheld for 12 h prior to the measurements. Of the study subjects, 11 (58%) used inhaled corticosteroids (ICS), and ten of these used ICS in combination with long-acting beta-agonists (LABA), whereas one used LABA without ICS. Nine subjects (47%) used long-acting anticolinergic (tiotropium), and none used short-acting anticolinergics. Two subjects used teophylline, and one subject used leukotriene receptor antagonist in addition to other medication. Nine subjects used SABA as rescue medication. Four subjects (21%) had no medication for COPD. Of the 19 patients, six had no comorbidities, ten patients received medication for hypertension, three for coronary heart disease, three for hypercholesterolemia, two for diabetes and two for rheumatoid arthritis. The 20 healthy subjects were recruited from the hospital staff and their relatives. None had a history or symptoms of respiratory diseases, and all were either life-time non-smokers or had smoked a maximum of five pack-years, but had stopped smoking at least 5 years ago. All subjects were free of respiratory infections for the preceding 4 weeks. The anthropometric and spirometric characteristics as well as data on the smoking history of the study population appear in Table 1.

**Table 1:** Characteristics of the populations studied.

	Healthy subjects (*n* = 20)	All COPD (*n* = 19)	COPD stage 2 (*n* = 12)	COPD stage 3–4 (*n* = 7)
Gender m/f (n)	5/15	13/6	7/5	6/1
Age, years	41 (23–58)	65 (54–72)	67 (54–72)	63 (55–69)
Height, cm	168·3 (153–184)	171·5 (151–190)	168·8 (158–180)	176·0 (151–190)
Weight, kg	70·8 (50–92)	82·6 (47–114)	87·8 (57–114)	73·7 (47–100)
Body mass index, kg m^−2^	24·9 (21·1–30·5)	28·1 (20·6–43·3)	30·8 (21·7–43·3)	23·5 (20·6–27·7)
FVC, l	4·4 (3·1–5·8)	3·5 (1·8–4·7)	3·3 (1·9–4·7)	3·7 (1·8–4·7)
FVC, % of predictedxy^a^	103·1 (86·5–135·5)	87·5 (67·8–125·2)	89·9 (72·2–125·2)	83·3 (67·8–95·4)
FEV1, l	3·5 (2·4–4·8)	1·7 (0·5–2·9)	1·9 (1·1–2·9)	1·3 (0·5–1·8)
FEV1, % of predicted^a^	98·6 (78·4–124·7)	53·0 (23·1–79·7)	62·5 (50·5–79·7)	36·6 (23·1–46·9)
FEV1/FVC	0·79 (0·72–0·92)	0·49 (0·28–0·66)	0·57 (0·34–0·66)	0·35 (0·28–0·41)
GOLD stage 2/3/4 (*n*)	NA	12/5/2	12/0/0	0/5/2
Pack years	NA	52 (20–100)	49 (20–100)	57 (30–100)

Parameters expressed as mean (range) unless otherwise stated.

COPD, chronic obstructive pulmonary disease; FVC, forced vital capacity; FEV1, forced expiratory volume in 1 s; GOLD, global initiative for chronic obstructive lung disease; NA, not applicable.

a Finnish reference values (Viljanen *et al.*, 1982).

### Study design

FENO was determined three times: at baseline, 10 min and 24 h after baseline. Spirometry was carried out on the first study day after the FENO measurements. The FENO measurements were taken in the Laboratory of Clinical Physiology and spirometry in the Research Unit for Pulmonary Diseases by experienced study nurses. This study was approved by the Ethics Committee of the Department of Medicine at the Helsinki University Central Hospital. All subjects provided their written informed consent.

### Fractional exhaled nitric oxide measurement

FENO was measured with a chemiluminescence analyser (Sievers 270B, Boulder, CO, USA) by using computer software specially developed for this purpose (Ekroos *et al.*, 2000). Two-point calibration of the analyser was performed daily before the FENO measurements. Expiratory airflow and exhaled volume were measured with a pneumotachograph (Baby Pneumotachograph, Erich Jaeger GmbH, Wurzburg, Germany) simultaneously with FENO in real time. The exhalation procedure fulfilled the criteria defined in the American Thoracic Society/European Respiratory Society guidelines on exhaled FENO measurements (ATS/ERS 2005). On both study days, the subjects rinsed their mouths with sodium bicarbonate solution (Hartwall Novelle®, Oy Hartwall Ab, Helsinki, Finland) prior to the first measurement, to eliminate any nitric oxide produced in the mouth. After the inhalation of synthetic NO-free air, subjects exhaled from total lung capacity with a flow rate of 50 ml s^−1^ against a flow resistor (model #7100R, 200 cmH_2_0 l^−1^ s^−1^, flow range 0–0·1 l s^−1^; Hans Rudolph, Shawnee, KS, USA). No nose clips were used. The subjects maintained the required flow rate with the aid of visual feedback from the computer screen. An acceptable measurement had a mean flow rate between 0·045 and 0·055 l s^−1^ and a duration of exhalation of at least 10 s. The mean value taken from a 3-s period during the end-exhaled NO plateau was recorded for analysis. At least three successive FENO measurements were taken, and their mean values were recorded for analysis. An acceptable coefficient of variation (CoV) for the successive FENO determinations was ≤10%.

### Spirometry

Spirometry was completed using a flow-volume device (VMax 20c; Sensor Medics, Yorba Linda, CA, USA) with the subject seated according to ATS/ERS 2005 criteria (Miller *et al.*, 2005; Pellegrino *et al.*, 2005). The spirometry variables analysed were FVC, FEV1 and FEV1/FVC. Published Finnish reference values were used (Viljanen *et al.*, 1982).

### Statistical analysis

Statistical analyses were performed with SPSS version 15.0 for Windows (SPSS, Chicago, IL, USA). FENO results are expressed as medians (25–75% quartiles), and non-parametric tests were applied, as FENO values are not normally distributed and the small number of measurements would otherwise be sensitive to the effect of single outliers. Bland–Altman plots served to illustrate the repeatability and intra-subject correlation of FENO measurements (Bland & Altman, 1986; Chinn, 1991); 95% of the differences between measurements were expected to lie within two standard deviations (SD). Intraclass correlation coefficients (ICC) with 95% confidence intervals were calculated, and ICC-values >0·6 were considered clinically significant (Faul *et al.*, 1999). The ICC is an application of analysis of variance that produces the measures of consistency or agreement of values within cases (Shrout & Fleiss, 1979; McGraw & Wong, 1996). Coefficients of variation (CoV) were calculated for intrasession and between-session repeatability by dividing the SD of the individual measurements by their mean, expressed as percentages. Wilcoxon’s pairwise test was used for paired observations. A *P*-value of <0·05 was considered statistically significant.

## Results

Of the 20 patients with COPD studied, 12 had moderate COPD [global initiative for chronic obstructive lung disease (GOLD) stage 2, FEV1 < 80% but ≥50%], five had severe COPD (GOLD stage 3, FEV1 < 50% but ≥30%) and three had very severe COPD (GOLD stage 4, FEV1 < 30%). One subject (woman, 73 years) with stage 4 disease was unable to perform technically acceptable FENO measurements (intrasession CoV was 10% at baseline, 18% at 10 min and 19% at 24 h) and thus was excluded from the analysis. Another subject with stage 4 disease had a CoV of 11% at the +10-min measurement, with a CoV of ≤10% at the baseline and +24-h measurements; the subject was included in the analyses. One subject (woman, 54 years, stage 2 disease) did not return for the second study day, so +24-h FENO measurements are available from 18 subjects. All of the healthy subjects were able to produce technically acceptable FENO measurements.

**Figure 1:**
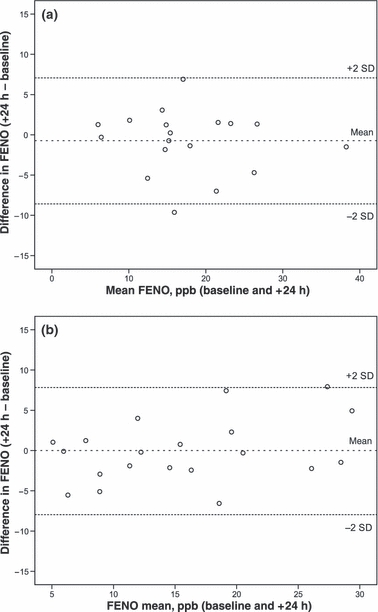
Bland–Altman analysis for the 24 h repeatability of fractional exhaled nitric oxide in (a) patients with chronic obstructive pulmonary disease and (b) healthy subjects.

Median FENO values for each session (baseline, +10 min and +24 h), CoV and ICC between baseline and +10-min measurements as well as between baseline and +24-h measurements for healthy subjects, for all patients with COPD and separately for COPD patients with stage 2 and stage 3–4 disease appear in Table 2. Intrasession repeatability for patients with COPD and healthy subjects ranged from 5·5% to 6·9%. The between-session repeatability (baseline and +24-h measurements) of FENO in patients with COPD and in healthy subjects is presented by Bland–Altman plots (Fig. 1a,b), where the mean of the baseline and +24-h FENO values are plotted against the difference between the FENO values of the two respective sessions.

**Table 2:** Median FENO values and their short-term variability in the groups studied.

	FENO, ppb baseline	FENO, ppb +10 min	CoV* (%)	ICC (95% CI)	FENO, ppb +24 h	CoV** (%)	ICC (95% CI)
Healthy (*n* = 20)	15·2 (10·1–21·6)	17·4 (10·8–26·4)	13·7	0·85 (0·67–0·94)	14·5 (7·7–22·3)	15·9	0·90 (0·77–0·96)
COPD (*n* = 19)	15·6 (12·8–22·5)	19·6 (15·8–23·2)	11·0	0·90 (0·77–0·96)	15·7 (11·1–22·8)	12·4	0·88 (0·72–0·95)
GOLD stage 2 (*n* = 12)	18·2 (14·5–25·7)	20·0 (16·1–28·1)	10·9	0·91 (0·74–0·97)	15·5 (11·1–29·3)	13·7	0·89 (0·65–0·97)
GOLD stage 3–4 (*n* = 7)	13·6 (9·2–18·6)	18·4 (9·1–20·4)	11·2	0·92 (0·32–0·97)	15·9 (11·0–20·5)	10·5	0·83 (0·37–0·97)

FENO expressed as medians (25–27% quartiles).

FENO, fractional exhaled nitric oxide; CoV, coefficient of variance (*between baseline and +10 min, **between baseline and +24 h); ICC, intraclass correlation coefficient; CI, confidence interval; COPD, chronic obstructive pulmonary disease.

Median FENO at baseline was 15·6 ppb (25–75% quartiles 12·8–22·5) in patients with COPD and 15·2 ppb (10·1–21·6) in healthy subjects. In one patient with COPD, the baseline FENO, 39 ppb, exceeded the upper normal limit of 30 ppb (Rouhos *et al.*, 2008). No comparison of FENO levels between the studied groups was performed as the groups were not age- or gender-matched and all pulmonary medication, including ICS influencing FENO, was allowed. No significant difference appeared between FENO values at baseline and at +24 h among either patients with COPD or healthy subjects (*P* = 0·62 and *P* = 0·68, respectively). FENO values at the +10-min measurement (when the subjects did not rinse their mouths with sodium bicarbonate) in both COPD and healthy subjects were slightly but significantly higher than at the +24-h measurement (*P* = 0·002 and *P* = 0·03, respectively) or at baseline (*P* = 0·008 and *P* = 0·002).

## Discussion

The present study demonstrated that the repeatability of FENO between successive days in patients with stable COPD is equally good compared to that in healthy subjects. A coefficient of variation of 12·4% for the FENO measurements performed at a 24-h interval suggests that a change in FENO exceeding 24% is likely to reflect a minimum measurable change of FENO in COPD.

A coefficient of variation of 12·4% in patients with COPD for the FENO measurements performed at a 24-h interval is in agreement with the results of the study by Bhowmik *et al.* (2005), who reported a CoV of 13·1% for the short-term repeatability of FENO in a group of 79 patients with moderate to severe COPD, although the flow rate for the FENO measurements (5 l min^−1^) differed from current recommendations (ATS/ERS 2005), and the exact interval for repeated measurements was not mentioned. Brindicci *et al.* (2005) studied the day-to-day variation as well as the diurnal variation of FENO in eight COPD patients with stage 2 disease as part of a larger study of 81 subjects (non-smokers and smokers with or without airway obstruction) using a multiple flow technique. They reported a high degree of reproducibility (ICC 0·993 for a flow rate of 50 ml s^−1^) in FENO measurements for the whole study group and concluded that the results are applicable in COPD of differing severity. In the present study, the repeatability was equally good in patients with moderate or very severe obstruction, except for one patient with very severe COPD who was unable to maintain the flow rate required to produce repeated FENO recordings that would meet the requirements of an acceptable measurement. Similarly, 11 of the 98 patients originally included in Bhowmik’s study were unable to perform acceptable measurements. These subjects were older (mean age 71 years) and had a lower FEV1 (mean 0·84 l) than did those capable of producing technically acceptable measurements (Bhowmik *et al.*, 2005).

The day-to-day repeatability of FENO using a flow rate of 50 ml s^−1^ has been reported to be good in healthy subjects. Our study found an ICC of 0·90 in healthy subjects between FENO measurements performed 24 h apart to be equal to ICC of 0·94 reported by Kharitonov *et al.* (2003) in a group of ten healthy non-smoking adults. Repeatability in asthmatics has been reported to be equally good, with an ICC of 0·90 for FENO measurements performed with a 24-h interval in a group of ten adults with steroid-naive asthma (Kharitonov *et al.*, 2003).

The results of our study suggest that a change in FENO concentration exceeding 24% is likely to reflect a measurable change of in the inflammatory process in COPD. A recent study (de Laurentiis *et al.*, 2008) measuring FENO (flow rate 50 ml s^−1^) once a month over a period of 1 year in 59 patients with COPD (mean age 66 years and mean GOLD stage 2·6) found a significant correlation between individual exacerbation rate and FENO CoV, as patients with a FENO CoV of >40% during the year reported a twofold increase in exacerbation rate over that of patients with a FENO CoV of <40%.

Because a spirometric manoeuvre may temporarily reduce FENO (Silkoff *et al.*, 1999) and SABA may temporarily increase FENO (Silkoff *et al.*, 1999), SABA were withheld for 12 h before the FENO measurements, and spirometry was performed after the FENO measurements on the first study day. Mouthrinsing with sodium bicarbonate or chlorhexidine is known to slightly reduce FENO levels by eliminating any nitric oxide produced in the mouth (Zetterquist *et al.*, 1999; Marteus *et al.*, 2005), but the duration of this effect remains unknown. The subjects in the present study rinsed their mouths before the first FENO measurements on both study days, but the procedure was not repeated before the +10-min measurements. This may explain the slightly higher FENO values in the +10-min measurements than in the baseline or +24-h measurements, as experience from our laboratory suggests that the reduction in FENO because of mouthrinsing may disappear in <10 min (Piirilä P, Rouhos A, Sovijärvi A. R. A, unpublished data). The staging of the severity of COPD according to the GOLD criteria should be based on postbronchodilator FEV1.The present study, however, used prebronchodilator values for practical reasons. Because the patients had been diagnosed with COPD on average 5 years previously and were clinically stable with regular medication, a significant bronchodilator response would be unlikely. To obtain a patient population representative of a real-life situation, COPD patients with comorbidities were included as long as they were clinically stable. The groups studied were not age- or gender-matched, and healthy subjects were more often women and younger than patients with COPD. However, older disabled subjects with airflow obstruction performed FENO measurements as well as did the younger healthy ones. The study included only stable COPD patients: all but one had FENO within normal limits (≤30 ppb). Thus, the results are not directly applicable to COPD patients with elevated FENO or with unstable disease, but reflect the minimum measurable change of FENO in COPD.

Unlike patients with asthma, only a subset of patients with COPD benefit from treatment with ICS. In subjects with fixed or partially reversible airway obstruction, an elevated sputum eosinophil count or elevated FENO has been found to relate to an improvement in FEV1 following steroid treatment (Fabbri et al., 2003; Brightling et al., 2005; Zietkowski et al., 2005; Kunisaki et al., 2008;). FENO measurement can thus be useful in focusing steroid treatment to those most likely to benefit from it. Because FENO measured with the conventional technique reflects airway inflammation mainly in the larger airways, it thus may not fully represent the more peripheral inflammation present in COPD. This peripheral component of FENO, better detected by using a technique of multiple flow rates, has been shown to be insensitive to steroid treatment, however, and thus may be inapplicable when the purpose of FENO measurement is to identify possible steroid responders. High level of bronchial NO flux has been reported to relate to symptom relief and improvement in FEV1 after steroid treatment, whereas no such association was detected in relation to alveolar NO (Lehtimäki *et al.*, 2010).

The findings of the present study indicate that the short-term repeatability of FENO is equally good in patients with stable COPD as in healthy subjects, although in patients with very severe disease, difficulties may arise in maintaining the required flow rate. The results suggest that a short-term change in FENO values exceeding 24% is likely to reflect a minimum measurable change in COPD.
